# ZnO Nanoparticles Treatment Induces Apoptosis by Increasing Intracellular ROS Levels in LTEP-a-2 Cells

**DOI:** 10.1155/2015/423287

**Published:** 2015-08-03

**Authors:** Caixia Wang, Xiaoke Hu, Yan Gao, Yinglu Ji

**Affiliations:** ^1^Key Laboratory of Coastal Biology and Bioresource Utilization, Yantai Institute of Coastal Zone Research, Chinese Academy of Sciences, 17 Chunhui Road, Laishan District, Yantai 264003, China; ^2^University of Chinese Academy of Sciences, Beijing 100049, China; ^3^National Oceanographic Center, Qingdao 266071, China; ^4^College of Marine Life Science, Ocean University of China, Qingdao 266003, China

## Abstract

Owing to the wide use of novel nanoparticles (NPs) such as zinc oxide (ZnO) in all aspects of life, toxicological research on ZnO NPs is receiving increasing attention in these days. In this study, the toxicity of ZnO NPs in a human pulmonary adenocarcinoma cell line LTEP-a-2 was tested *in vitro*. Log-phase cells were exposed to different levels of ZnO NPs for hours, followed by colorimetric cell viability assay using tetrazolium salt and cell survival rate assay using trypan blue dye. Cell morphological changes were observed by Giemsa staining and light microscopy. Apoptosis was detected by using fluorescence microscopy and caspase-3 activity assay. Both intracellular reactive oxygen species (ROS) and reduced glutathione (GSH) were examined by a microplate-reader method. Results showed that ZnO NPs (≥0.01 *μ*g/mL) significantly inhibited proliferation (*P* < 0.05) and induced substantial apoptosis in LTEP-a-2 cells after 4 h of exposure. The intracellular ROS level rose up to 30–40% corresponding to significant depletion (approximately 70–80%) in GSH content in LTEP-a-2 cells (*P* < 0.05), suggesting that ZnO NPs induced apoptosis mainly through increased ROS production. This study elucidates the toxicological mechanism of ZnO NPs in human pulmonary adenocarcinoma cells and provides reference data for application of nanomaterials in the environment.

## 1. Introduction

With rapid development of nanotechnology, the application field and commercial manufacturing scale of synthetic nanomaterials and nanoparticles (hereinafter referred to as NPs) have undergone significant expansion worldwide. This situation has increasingly aggravated the damage to ecological environment and human health, mainly because various NPs have diverse effects (small-scale, surface, quantum-size, and/or macroscopic quantum tunneling) [1]. Research of nanomaterial toxicology is presently at an early development stage. Associated research has been conducted on carbon nanomaterials first, and the test objective has been extended from mouse [2] to aquatic organisms (largemouth bass,* Daphnia magna*,* Tetrahymena thermophila*, and crucian carp) and human cells [3]. Therefore, the biological safety of NPs has aroused great concerns by governments and academic circles.

Metal oxide nanomaterials such as zinc oxide (ZnO) NPs exhibit antibacterial, anticorrosive, antifungal, and UV-filtering properties as well as certain cytotoxicity [1]. Compared to titanium dioxide (TiO_2_) NPs, ZnO NPs exert relatively strong toxic effects on human pulmonary epithelial cells, and the toxicities of both kinds of metal oxide NPs are controlled by their physicochemical characteristics (e.g., size and crystal phase) [3]. Regarding the underlying mechanism of toxicity, TiO_2_ NPs promote the generation of intracellular reactive oxygen species (ROS) by modulating cell metabolism with light [4], whereas overproduction of ROS may damage the antioxidant mechanism in macrophages [5] and cause toxic effects in brain microglia or other cells [6, 7]. Similarly, ZnO NPs may cause oxidative stress in macrophages and human cells, resulting in lipid peroxidation, cell membrane damage, and ultimately cell death or apoptosis [8, 9]. Despite previous research achievements, the toxicological mechanism of ZnO NPs has not been elucidated in certain species or cancer cells. Exploring the exact mechanism of this novel nanomaterial is of great value for clinical trials of cancer treatment.

In the present study, we assessed the* in vitro *toxicity of ZnO NPs in a human pulmonary adenocarcinoma cell line, LTEP-a-2. Log-phase cells were exposed to different concentrations of ZnO NPs for hours, followed by* in vitro *tests of cell viability, survival rate, morphological changes, apoptosis, and intracellular ROS and reduced glutathione (GSH). The results were analyzed to explore the toxicological mechanism of ZnO NPs in LTEP-a-2 cells, further laying a foundation for in-depth toxicological study and clinical trials of this nanomaterial for cancer treatment.

## 2. Materials and Methods

### 2.1. ZnO NPs

All experiments were carried out on an ultraclean bench to prevent interference of external factors. Highly purified (99.9%) ZnO NPs were purchased from Sigma Aldrich (St. Louis, USA). Stock solutions of ZnO NPs were prepared in Dulbecco's modified Eagle's medium (DMEM) containing 50 *μ*g/mL fetal bovine serum (FBS). To avoid particle aggregation, the prepared solutions were sonicated three times (20 s/time) prior to use [10, 11]. ZnO NPs in DMEM were characterized in terms of morphology, diameter, tendency of aggregation, and intracellular distribution using a scanning electron microscope (SEM, Hitachi S-4800, Japan). Zeta potential analysis of ZnO NPs in DMEM was performed by using dynamic light scattering (Malvern Zetasizer ZS9, Worcestershire, UK).

### 2.2. Cell Culture

Human pulmonary adenocarcinoma cells LTEP-a-2 were obtained from China Center for Type Culture Collection (Wuhan, China) and maintained in DMEM cell culture medium (Gibco, Grand Island, NY, USA) supplemented with 10% FBS, 100 U/mL penicillin, and 100 *μ*g/mL streptomycin (37°C, 5% CO_2_). For each of the following tests, an aliquot of log-phase culture broth was taken and diluted to obtain the density of 10^5^-10^6^ cells/mL.

### 2.3. Cell Viability Assay

The viability of LTEP-a-2 cells was assayed by using the 3-(4,5-dimethylthiazol-2-yl)-2,5-diphenyltetrazoliumbromide (MTT) method [12]. Log-phase cells were harvested and thoroughly washed with phosphate-buffered saline (PBS) and then inoculated into 96-well plates (Nunc, Roskilde, Denmark). When the cell density reached approximately 5 × 10^4^ cells/well, different concentrations of ZnO NPs (0, control; 0.01, 0.25, 0.5, 1.0, and 1.5 *μ*g/mL) were added into triplicate wells for 4, 8, 12, and 24 h of exposure. After aspirated incubation, a medium containing 20 *μ*L of 5 mg/mL MTT was added and the culture was continuously incubated. Four hours later, blue formazan crystal appeared at the bottom of wells, which was then dissolved with 150 *μ*L dimethyl sulfoxide. Cell viability was detected by measuring the absorbance of cell culture broth at 490 nm using a microplate reader (Thermo Varioskan Flash 3001, USA).

### 2.4. Trypan Blue Exclusion Test

The lethality of ZnO NPs on LTEP-a-2 cells was assessed by the trypan blue exclusion test [13]. Cells were seeded in 6-well plates with different concentrations of ZnO NPs (0, control; 0.05, 0.1, 0.2, 1.0, and 5.0 *μ*g/mL) for 12 h of exposure in a humidified incubator (5% CO_2_, 37°C). Thereafter, cells were trypsinized and resuspended in equal volumes of culture medium and trypan. Viable (unstained) and nonviable (blue-stained) cells were counted using a haemocytometer to calculate the total numbers of living and dead cells.

### 2.5. Morphological Assay

LTEP-a-2 cells were cultured in 6-well plates with different concentrations of ZnO NPs (0, control; 0.01, 0.05, 0.1, 0.2, and 0.5 *μ*g/mL) for 4 h of exposure and then fixed with methanol and dried. The cells were stained for 20 min with Giemsa staining solution, rinsed in deionized water, air-dried, and examined under an optical microscope (SH-60, Olympus, Japan) equipped with a digital camera [14]. The stained cells were examined in terms of size, regularity of the margin, and morphological characteristics of the nucleus.

### 2.6. Apoptosis Detection

ZnO NPs-induced apoptosis after 4 h of exposure was detected by acridine orange/ethidium bromide (AO/EB) double staining. Cells were stained with 100 *μ*g/mL AO/EB (Sigma, USA) for 2 min, followed by examination using a fluorescence microscope (Leica DM 5000B, Leica Microsystems, Germany). The detection criterion is that normal cells present uniform green nuclei, and late apoptotic cells present orange to red nuclei with condensed or fragmented chromatin [15, 16].

### 2.7. Caspase Activity Assay

Caspase activity was assayed according to the method of Vyas et al. [17]. Cells were cultured in 96-well plates with indicated concentrations of ZnO NPs for 4 h and then harvested by centrifugation at 1000 ×g for 10 min. The activity of caspase-3 was detected by using a colorimetric assay kit (Nanjing Jiancheng Bioengineering Institute, Nanjing, China). Cells were washed with PBS and resuspended in five volumes of lysis buffer (20 mmol/L Hepes, pH 7.9; 20% Glycerol; 200 mmol/L KCl; 0.5 mmol/L EDTA; 0.5% NP40; 0.5 mmol/L DTT; and 1% protease inhibitor cocktail). The content of protein was measured by using the Bradford method, and the absorption of cell culture broth at 405 nm was measured using a microplate reader (Infinite M200, Tecan, Switzerland) [18]. All treatments were performed in triplicate.

### 2.8. Intracellular ROS Assay

The intracellular ROS level was measured by active oxygen detection [19–21]. H2DCFDA was deacetylated intracellularly by using a nonspecific esterase and then oxidized by cellular peroxides, yielding a fluorescent compound, 2,7-dichlorofluorescein (DCF, *λ*EX/*λ*EM = 485 nm/535 nm). Cells were treated with indicated concentrations of ZnO NPs (0, control; 0.01, 0.05, 0.5, 1.0, and 1.5 *μ*g/mL) for 4 h and then washed with PBS and incubated in 30 *μ*mol/L H_2_DCFDA at 37°C for 30 min. The content of DCF was detected by using a microplate reader (Varioskan Flash 3001, Thermo, USA). Each group was maintained with the same number of cells in triplicate.

### 2.9. Intracellular GSH Content Assay

The intracellular GSH content was determined by using a microplate-reader method with a commercial kit (Nanjing Jiancheng Bioengineering Institute, Nanjing, China). LTEP-a-2 cells were inoculated into 6-well plates at 10^6^cells/well and then exposed to different concentrations of ZnO NPs (0, control; 0.01, 0.05, and 0.25 *μ*g/mL) for 4 h. Cells were then harvested and washed with PBS. The content of GSH was assayed by measuring the absorbance of cell extract at 412 nm using a microplate reader, calculated according to a standard curve, and normalized by the protein concentration detected using the Bradford method (Sangon, Shanghai, China) [22].

### 2.10. Statistical Analysis

All experimental data are presented as means ± standard error of the mean from at least three independent experiments. Data comparison between treatments was accomplished by one-way analysis of variance and Student's *t*-test (*P* < 0.05 considered statistically significant). Statistical analysis was performed in SPSS16.0 (SPSS Inc., USA) and Origin 6.0 (OriginLab Corp., USA).

## 3. Results and Discussion

### 3.1. Characteristics of ZnO NPs

**Figure 1:**
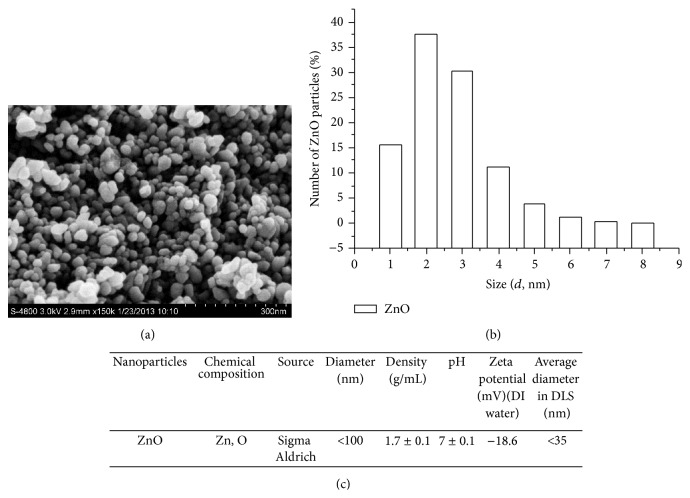
Major characteristics of ZnO nanoparticles used in this study. (a) Scanning electron micrograph, (b) size distribution, and (c) major physical properties.

A description of the morphology and physicochemical properties of ZnO NPs is regarded as a comparative study in the field of cytotoxicity research [23, 24]. In the present study, SEM image shows that the ZnO NPs in use are mainly anxiolytic shaped and are partially rhombic (Figure 1(a)). Mean grain diameter of the ZnO NPs is 30 ± 5 nm, which matches the supplier's declaration. Zeta potential data indicate that the ZnO NPs have a positive surface charge, −18.6 mV at pH 7.4 in DMEM (Figure 1(b)), which is inadequate to stabilize the suspension of ZnO NPs via repulsive force and thus may cause NPs aggregation in DMEM. The size distribution of ZnO NPs in DMEM, as determined by dynamic light scattering, shows great variations (Figure 1(c)).

### 3.2. Cytotoxicity of ZnO NPs

**Figure 2:**
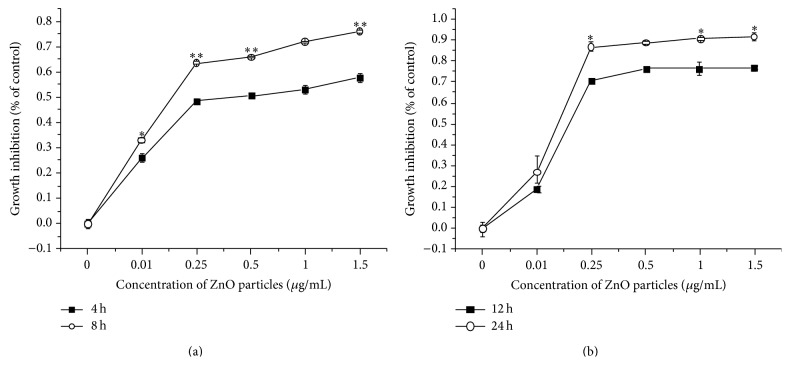
Relative viability of LTEP-a-2 cells after 4–24 h of exposure to different concentrations of ZnO nanoparticles (0 *μ*g/mL, control). (a) 4 and 8 h and (b) 12 and 24 h. ∗ versus control, *P* < 0.05; ∗∗ versus control, *P* < 0.01 by Student's *t*-test.

The cytotoxicity of ZnO NPs in LTEP-a-2 cells was tested by MTT assay using a protocol adopted from previously published reports and manufacturer's instructions [9, 25, 26], expressed as the percentage of cell mortality relative to the control treatment (Figure 2). After 4–24 h of exposure to ZnO NPs (0.01–1.5 *μ*g/mL), cell viability declined substantially in a concentration- and time-dependent manner; the declines were especially significant after 8 h of exposure to ZnO NPs ≥ 0.25 *μ*g/mL (*P* < 0.05). The number of cell deaths among all these doses has been nearly 20% higher than the lower doses over the past 24 h. High cytotoxicity can be observed in cells treated with ZnO NPs when compared to control group. These results indicate that cell proliferation was inhibited significantly with increasing concentration of ZnO NPs.

### 3.3. ZnO NPs Reduced Cell Survival Rate

**Figure 3:**
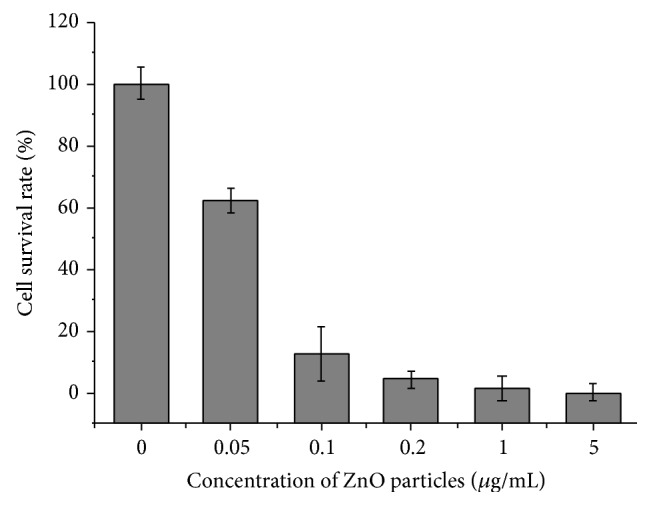
The survival rate of LTEP-a-2 cells detected by trypan blue exclusion test after 12 h of exposure to different concentrations of ZnO nanoparticles (0 *μ*g/mL, control).

The dye exclusion test was used to determine the number of viable cells present in a cell suspension. This method is based on the principle that live cells possess intact cell membranes that exclude certain dyes, such as trypan blue, eosin, or propidium, whereas dead cells do not [27]. In this test, a cell suspension was simply mixed with dye and then visually examined to determine whether cells take up or exclude dye. A viable cell was identified with a clear cytoplasm and a nonviable cell with a blue cytoplasm. Results showed that after 12 h of exposure to ZnO NPs (0.05–5.0 *μ*g/mL), the survival rate of LTEP-a-2 cells underwent substantial decreases in a concentrate-dependent manner (Figure 3). In the presence of low concentration of ZnO NPs (0.05 *μ*g/mL), cell survival rate remained above 60%, showing a nearly 40% decrease relative to the control treatment; as the concentration of ZnO NPs was increased to 0.1 *μ*g/mL, cell survival rate underwent another 40% decrease, down to approximately 20% only. Together, these results confirm that the presence of ZnO NPs significantly affected cell survival even at low concentrations (e.g., 0.05–0.1 *μ*g/mL).

### 3.4. ZnO NPs Induced Morphological Changes

**Figure 4:**
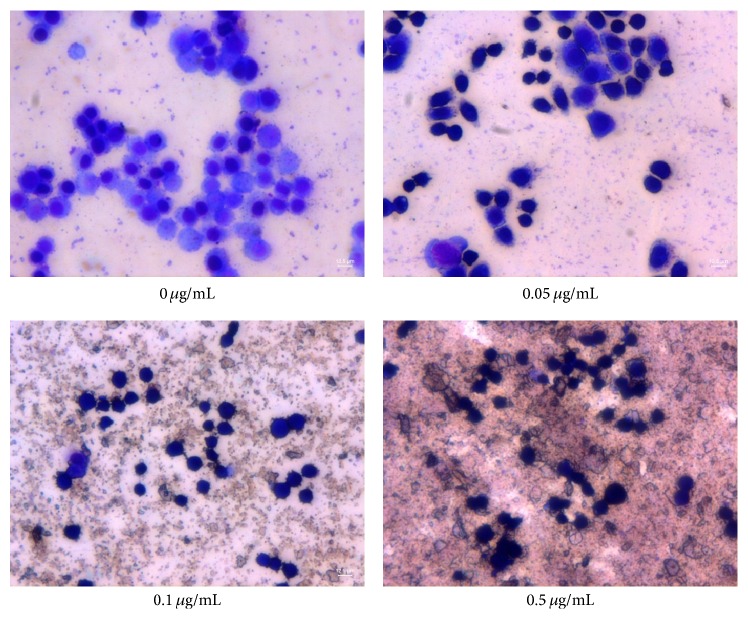
ZnO nanoparticles-induced morphological changes in LTEP-a-2 cells examined by Giemsa staining. Note significant changes in cells after 4 h of exposure to ZnO NPs (0 *μ*g/mL, control).

Giemsa staining is commonly used for identifying morphological changes of monocytes/macrophages in cell preparation [28]. In the present study, Giesma staining was used to examine ZnO NPs-induced morphological changes in LTEP-a-2 cells for further characterizing the cytotoxicity of this nanomaterial. Microscopic examinations revealed that Giemsa-stained control cells (0 *μ*g/mL ZnO NPs) predominantly had round regular cell margins and large nuclei (Figure 4); that is, the control cells were associated with rapid DNA synthesis and fast proliferation. With increasing concentrations of ZnO NPs (0.05, 0.1, and 0.5 *μ*g/mL), there were evident increases in the number of pyknotic shrinking cells following a dose-dependent manner (Figure 4); the increasing morphological changes in the presence of ZnO NPs coincided well with the declines in cell survival rate (Figure 3); thus they were associated with proliferation inhibition and/or cell death.

### 3.5. ZnO NPs Induced Apoptosis

**Figure 5:**
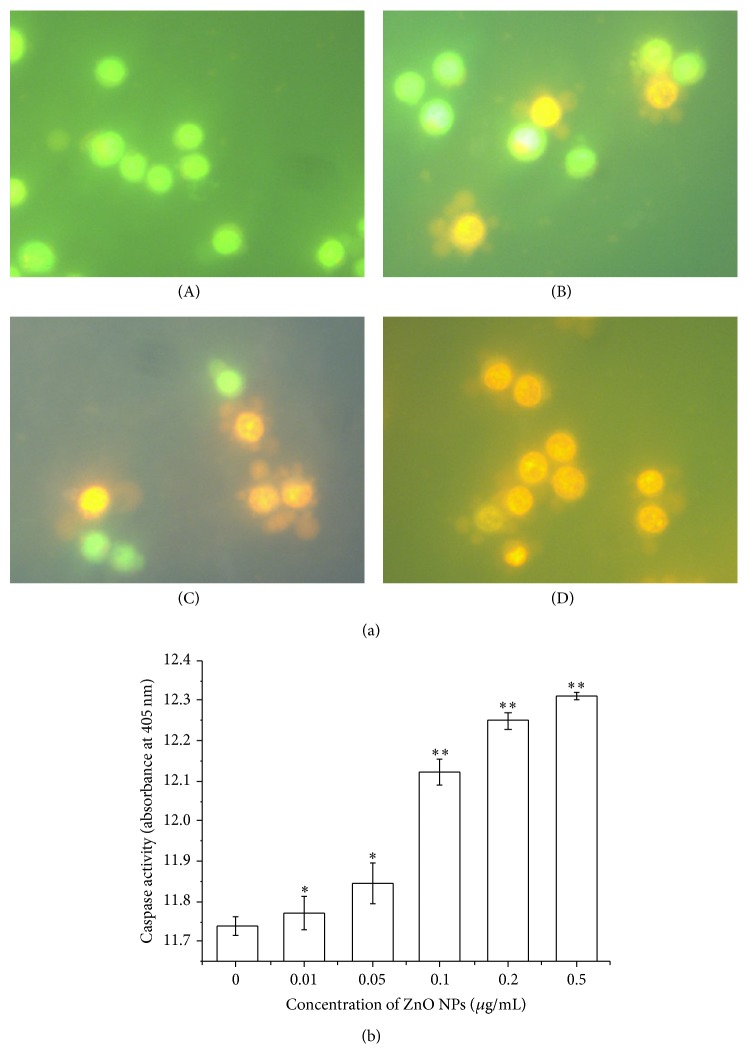
ZnO nanoparticles-induced apoptosis in LTEP-a-2 cells after 4 h of exposure. (a) Morphologic examination of LTEP-a-2 cells by AO/EB fluorescence staining ((A) 0 *μ*g/mL, control, (B) 0.05 *μ*g/mL, (C) 0.1 *μ*g/mL, and (D) 0.2 *μ*g/mL) and (b) caspase-3 activity. ∗ versus control, *P* < 0.05; ∗∗ versus control, *P* < 0.01 by Student's *t*-test.

AO/EB staining is considered an ideal method for distinguishing apoptotic cells from necrotic ones [29, 30]. Here we use AO/EB staining to verify whether ZnO NPs inhibit proliferation of LTEP-a-2 cells by inducing apoptosis or killing cells directly (necrosis). During morphologic examinations, normal viable LTEP-a-2 control cells were stained green (0 *μ*g/mL ZnO NPs), and the apoptotic cells exposed to ZnO NPs (0.05, 0.1, and 0.2 *μ*g/mL) appeared as bright green arcs in an early stage and with condensed, yellow/orange nuclei in the late stage (Figure 5(a)). Additionally, the results of caspase-3 activity assay showed that exposure of ZnO NPs induced significant increases in caspase-3 activity compared to the control treatment (*P* < 0.05 for 0.01–0.05 *μ*g/mL ZnO NPs, *P* < 0.01 for 0.1–0.5 *μ*g/L ZnO NPs; Figure 5(b)). As a key mediator, caspase-3 plays a pivotal role in caspase-dependent apoptosis [31]. Together, these results confirm that exposure of ZnO NPs induced substantial apoptosis in LTEP-a-2 cells even at low concentrations (e.g., 0.01 *μ*g/mL), thus inhibiting cell proliferation.

### 3.6. ZnO NPs Increased Intracellular ROS

**Figure 6:**
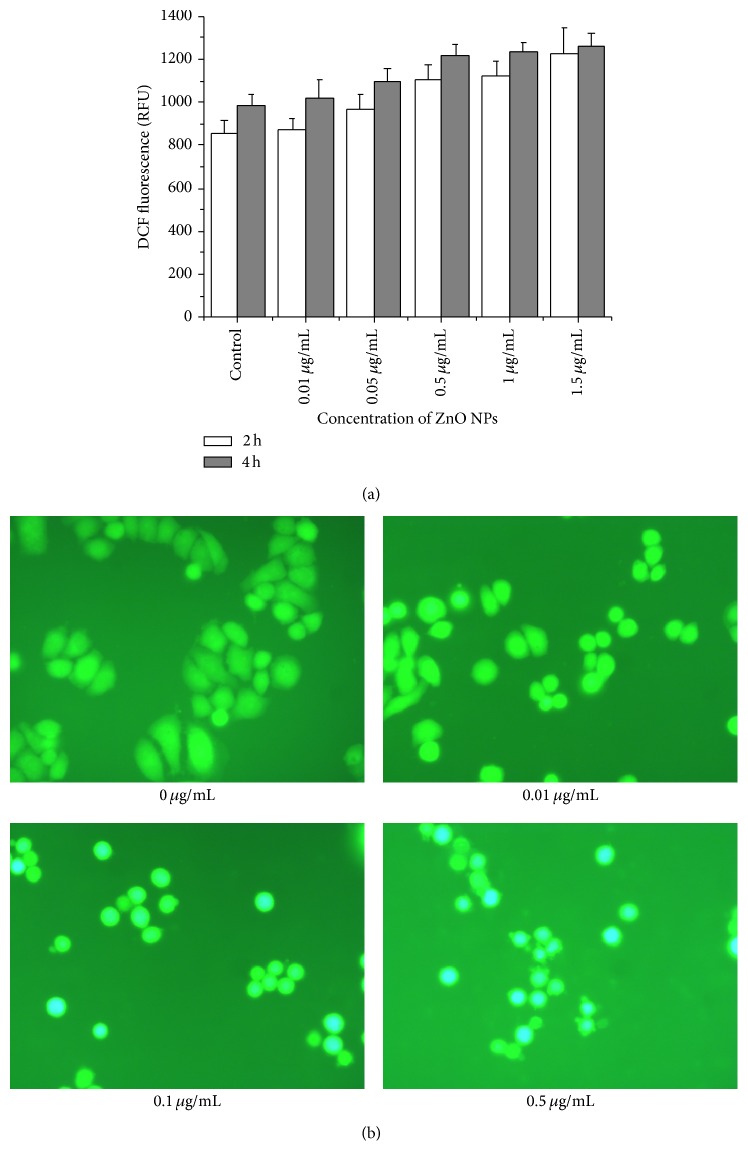
Increased production of intracellular reactive oxygen species (ROS) in LTEP-a-2 cells after 4 h of exposure to ZnO nanoparticles. (a) ROS level determined via spectrophotometry and (b) fluorescence intensity measured by microscopy.

Oxidative stress is considered one of the causative factors of apoptosis in pathogenesis and aggressiveness of most cancers [32]. A moderate rise in ROS level often induces cell proliferation whereas excessive amounts of ROS induce apoptosis [33]. To clarify the mechanism through which ZnO NPs induce apoptosis in LTEP-a-2 cells, we determined the intracellular ROS level by measuring the oxidation of nonfluorescent DCFH-DA to its highly fluorescent derivative DCF. Results showed that ZnO NPs stimulated ROS formation in cells following a concentration-dependent manner (Figure 6(a)). Under a fluorescence microscope, strong green fluorescence was observed in LTEP-a-2 control cells, whereas blue fluorescence was observed in cells after exposure to ZnO NPs. With increasing concentrations of ZnO NPs, the blue fluorescence was greatly strengthened accompanied by the appearance of apoptosis vesicles (Figure 6(b)). Together, these results demonstrate that ZnO NPs induce apoptosis in LTEP-a-2 cells through increased production of ROS, consistent with previous findings in macrophages and human liver cells [8, 9], as well as* in vivo* and* in vitro* tests of a wide range of NPs species [34, 35].

### 3.7. ZnO NPs Decreased Intracellular GSH Content

**Figure 7:**
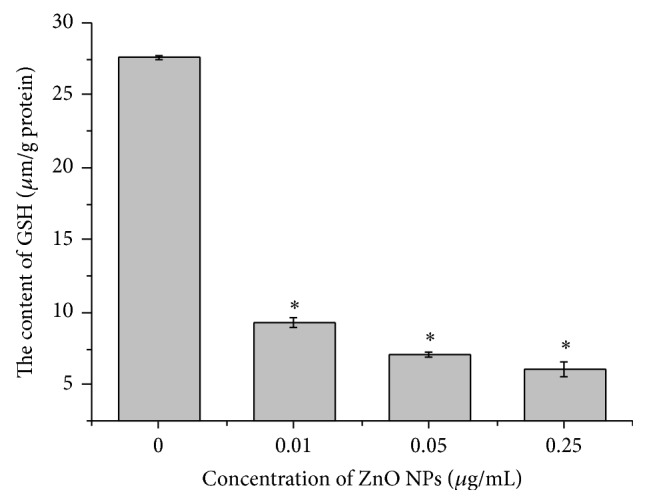
Depletion of intracellular glutathione (GSH) in LTEP-a-2 cells after 4 h of exposure to ZnO nanoparticles (0 *μ*g/mL, control). GSH content determined by spectrophotometry. ^*^ *P* < 0.05, ^**^ *P* < 0.01 versus control by Student's *t*-test.

GSH is one of the most abundant intracellular antioxidant thiols, which is involved in cell redox homeostasis and is central to defensive mechanisms against toxic agents and oxidant-mediated injury [36, 37]. In the present study, the GSH content significantly declined in LTEP-a-2 cells exposed to ZnO NPs (0.01–0.25 *μ*g/mL) for 4 h compared with the control cells (*P* < 0.05, Figure 7). The depletion of GSH coincided with the enlarging tendency of intracellular ROS level (Figure 6) once again demonstrating that ZnO NPs damaged the antioxidant mechanism of LTEP-a-2 cells.

## 4. Conclusions

In recent decades, nanomedicine has attracted considerable attention in the field of medicine [38–41]. Despite the advantages of nanotechnology, appropriate security measures should be taken to prevent potential hazardous effects of NPs. In the present study,* in vitro* test results show that ZnO NPs imposed distinct toxic effect on LTEP-a-2 cells, and the declines in cell viability and survival rate coincided with specific morphological changes and the occurrence of apoptosis. Further exploration of the toxicological mechanism revealed that the increase in ROS coincided with depletion of GSH in apoptotic cells, suggesting that oxidative stress may be the primary toxicological mechanism of ZnO NPs in LTEP-a-2 cells. As a common mechanism for NPs-induced cell oxidative damage, increased ROS generation has been confirmed by* in vivo* and* in vitro* tests of a wide range of NPs species. Oxidative stress often leads to cell death, by either apoptosis signaling pathways or necrosis signaling pathways depending on its extent of severity. With increasing evidence for the toxicity of NPs, it is important to plan out precautionary measures and to prevent human exposures to NPs.
